# Teaching and Assessing ED Handoffs: A Qualitative Study Exploring Resident, Attending, and Nurse Perceptions

**DOI:** 10.5811/westjem.2015.8.27278

**Published:** 2015-10-22

**Authors:** Moira Flanigan, James A. Heilman, Tom Johnson, Lalena M. Yarris

**Affiliations:** Oregon Health and Science University, Department of Emergency Medicine, Portland, Oregon

## Abstract

**Introduction:**

The Accreditation Council for Graduate Medical Education requires that residency programs ensure resident competency in performing safe, effective handoffs. Understanding resident, attending, and nurse perceptions of the key elements of a safe and effective emergency department (ED) handoff is a crucial step to developing feasible, acceptable educational interventions to teach and assess this fundamental competency. The aim of our study was to identify the essential themes of ED-based handoffs and to explore the key cultural and interprofessional themes that may be barriers to developing and implementing successful ED-based educational handoff interventions.

**Methods:**

Using a grounded theory approach and constructivist/interpretivist research paradigm, we analyzed data from three primary and one confirmatory focus groups (FGs) at an urban, academic ED. FG protocols were developed using open-ended questions that sought to understand what participants felt were the crucial elements of ED handoffs. ED residents, attendings, a physician assistant, and nurses participated in the FGs. FGs were observed, hand-transcribed, audio-recorded and subsequently transcribed. We analyzed data using an iterative process of theme and subtheme identification. Saturation was reached during the third FG, and the fourth confirmatory group reinforced the identified themes. Two team members analyzed the transcripts separately and identified the same major themes.

**Results:**

ED providers identified that crucial elements of ED handoff include the following: 1) Culture (provider buy-in, openness to change, shared expectations of sign-out goals); 2) Time (brevity, interruptions, waiting); 3) Environment (physical location, ED factors); 4) Process (standardization, information order, tools).

**Conclusion:**

Key participants in the ED handoff process perceive that the crucial elements of intershift handoffs involve the themes of culture, time, environment, and process. Attention to these themes may improve the feasibility and acceptance of educational interventions that aim to teach and assess handoff competency.

## INTRODUCTION

In order to address medical errors made during transitions of care, multiple regulatory agencies, including the Accreditation Council for Graduate Medical Education and the Joint Commission on Accreditation of Healthcare Organizations, have called for guidelines to ensure resident competency in performing safe, effective handoffs.^1^,^2^ Successful implementation of a standardized educational intervention to improve handoffs has recently been shown to reduce medical errors in the inpatient pediatric setting.^3^ In the emergency department (ED), several studies and consensus statements have begun to identify the core elements of ED provider handoffs.^4^–^11^ Although understanding what makes ED handoffs unique is crucial from an operational perspective, this core content knowledge does not alone shed light upon the best way to teach and assess handoff practices in the ED setting.

Educational interventions must go beyond presenting core content in order to translate knowledge into performance improvement–learners must also be receptive to the intervention and incorporate the content and process into their practice. A first step of designing instructional materials is to describe the problem that the intervention aims to address, and assess the perceptions and needs of the learner, as well as the important stakeholders who will interact with the learner in the clinical environment.^12^ Although the literature describes key elements of ED handoffs, we identified a knowledge gap in understanding how the perceptions of providers’ needs in the ED handoff process may impact the success of interventions designed to teach and assess ED handoffs.

To explore provider perceptions of ED handoffs that may inform or impact educational interventions aiming to improve provider performance, we designed a qualitative study to explore two central questions: 1) What themes emerge when exploring nurse, resident, attending, and midlevel provider perceptions regarding ED handoffs? and 2) What interprofessional and cultural processes take place during the ED handoff process? Using this analytical background, our study aimed to identify the crucial elements of ED-based handoffs that may be barriers to developing and implementing successful educational interventions to teach and assess ED handoff competency.

## METHODS

### Settings and Participants

This qualitative study of provider perceptions of ED handoffs was conducted in an urban, tertiary-care, academic ED with approximately 50,000 patient visits per year. Our three-year emergency medicine residency program, comprised of 33 residents and 24 core faculty, provides 24-hour resident coverage in the ED. Residents, attendings, charge nurses, and occasionally midlevel providers contribute to a resident-led handoff at each change of shift. All residents, attendings, midlevel providers, and nurses in the ED were invited to participate in the study by email invitation. Two of the four focus groups (FGs) were composed of a mixed group of residents, attendings and charge nurses. One of these FGs included a physician assistant. The other two FGs included only residents and attendings. FG size ranged from four to eight individuals and each group had participants who had not previously participated. Participation was voluntary and confidential. This study was approved by our institutional review board.

### Study Protocol

This study was a pre-planned separate phase of a larger study that aimed to adapt a standardized handoff process to the ED setting. We used a grounded theory approach and constructivist/interpretivist paradigm that sought to understand the perceptions of the various care providers in the ED.^13^–^16^ Our approach applied an iterative process, theoretical sampling, and a constant comparative method of data analysis. The primary phenomenon that we aimed to explore–the current intershift handoff–was studied by eliciting interprofessional perceptions regarding its standardization, safety, efficiency, and factors that may impact efforts to teach and assess handoff competency in ED providers. Our study team members included ED attendings, residents, and a student volunteer. Because multiple study investigators were known to the participants, and already a part of the handoff culture, a member of the team (MF) who was not known to the participants and was new to the culture was trained and led the facilitation of all FGs. We chose a theoretical sampling strategy that was purposive in that we sought to recruit groups of interprofessional providers representative of the providers who are actively engaged in handoffs in our institution. Subsequent theoretical sampling was guided by the categories and concepts that emerged in initial data collection, in order to maximize our understanding of relationships between concepts and developing themes. For example, the first FG did not include a midlevel provider, and early data analysis suggested that the midlevel provider voice may lend crucial insight into the handoff phenomenon. Therefore, a midlevel provider was recruited for a subsequent FG.

### FG Protocol

The FG protocol for this study phase was created simultaneously with the portion of the protocol that aimed to inform standardization ED handoff practices. Open-ended questions were developed that sought to understand what participants felt were the crucial elements of ED handoffs. The discussions were allowed to proceed organically, and the facilitator probed as necessary to explore factors relevant to understanding the barriers and promoters of effective ED handoffs. Participants were prompted to rely on their cumulative experiences in all the EDs in which they had worked so that themes would have increased external validity and not be institution-specific. Three primary FGs were conducted in October 2014; each was observed, audio-recorded, and hand-transcribed. The study team conducted ongoing data analysis to determine that saturation was reached after the third FG. A fourth confirmatory FG was held, which did not reveal additional themes.

### Data Analysis

Data analysis began with the hand transcription of FG proceedings during the FGs on poster paper, and then subsequent transcriptions of the audio-recordings by the MF. We anonymized and de-identified participant data. Data were then separately analyzed and coded by two team members using an iterative process of code categorization, concept identification, and constant comparative theme and subtheme identification. In order to assure trustworthiness and credibility of data analysis, member checking was performed at the conclusion of each FG by directed group review of the data scribed onto poster paper during the session. We performed triangulation by comparing FG transcripts with observer notes and hand-transcribed session notes.

## RESULTS

Analysis of FG data demonstrated three major categories that contribute to the collective conceptual understanding of the ED provider handoff: 1) the ontological framework; 2) cultural expectations; and 3) environmental factors specific to the ED setting (Figure). FG participants’ perceptions revealed four dominant themes: Culture (the ability of a new educational process to change existing cultural expectations and norms, or the overall efficacy of implementation in the face of those cultural norms), Time (as seen in a collective desire for shortened, yet effective, processes and the general reticence for processes that may elongate the formal handoff procedure); Environment (how the physical location of the sign-out affects participants’ learning experience, as well as the physician-patient relationship); and Process (information flow and order, consensus building). The Table illustrates the themes, subthemes, representative quotes, and educational considerations that emerged from analyzing our participants’ perceptions.

### Culture

An underlying culture marked by individuality, attending-resident hierarchy, and unyielding norms was alluded to throughout all FGs. Cultural reticence towards standardization in part centered on perceived lost individuality, related to both personal preference for handoff style as well as individual learning style and ability. Individuality also contributed to a complex attending-resident dynamic, in which instruction is not always based on standardized format or learner needs but individual attending preference, which may lead to a high degree of variability and quality in information communicated. Overall, this nebulous educational format discomfited providers. Unfamiliarity with systems, both existing and new, was a running subtheme that threaded its way through FGs, connected to individuality, fear of change, and cultural expectations and norms that allowed for a high degree of variability. Yet, despite these underlying identified motifs, ED culture was still viewed as a process that could be changed. In fact, providers perceived its potential to evolve, and that educational interventions could be successfully implemented if both individual growth and changing collective expectations were considered. Although interprofessional providers expressed concerns about the current system, they also expressed willingness to adopt new practices and a desire to work together towards a shared goal of improved ED handoff outcomes.

### Time

For many participants, time–especially its perceived scarcity–was an integral factor in willingness to embrace a new operational practice. Participants expressed frustration with handoffs perceived to be extraneous or elongated, especially due to interruptions. This desire for brevity also was identified as a potential threat towards provider acceptance of new educational interventions, in that providers may be reticent to buy-in to a new process if it is perceived as too cumbersome or poorly implemented.

### Environment

Providers’ perceptions of current handoff practices and their willingness to accept future interventions were intricately tied to the environment of the handoff process. Concerns regarding the physical location of the ED sign out were three-fold. First, providers expressed that the lack of an officially designated and consistent location for all handoffs contributed to a poor understanding of when handoff was in progress, and was implicitly linked to frequent and unnecessary interruptions during the handoff process. Participants voiced a perception that a designated handoff location might decrease the frequency of interruptions, and improve patient safety and privacy. Second, although a number of resident and attending participants cited that a non-designated space leads to an increased number of interruptions by ED staff, nurse participants voiced the experienced reality that isolated areas allow sequestration and a disconnect between interprofessional providers in different roles. The third concern was related to location’s effect on provider-patient interactions and relationships. Participants expressed that the current practice of conducting the handoff at a central computer station lacked privacy, not only for participating teams but also for patients. A number of participants advocated for bedside handoffs, as an in-person location was also perceived as providing both effective patient care and a better learning opportunity.

### Process

Underlying provider concerns regarding time, location, and culture were perceptions of individual and collective conceptual understanding, and the processes by which these are formed. Conceptual understanding, as espoused throughout the FGs, encompasses how both a single participant receives and synthesizes information (individual conceptual understanding), and how a group interacts with this individual comprehension to collaborate, build consensus, and in turn influence individual thought. Many providers describe this process in terms of order; information is synthesized based on the order in which it is received. Although order was not typically associated with whether or not a system would be implemented successfully, its consistent presence as a vital aspect of the ideal handoff emphasizes the impact this factor has, and how it is impacted in turn by the other identified factors of culture, time, and location.

## DISCUSSION

Understanding the three major categories that contribute to the collective conceptual understanding (Figure) of the ED provider handoff has important implications for emergency medicine resident education. The themes identified that create this framework were clearly separate entities in their specifics, yet the categories were also deeply intertwined with each other. Appreciating this interconnectedness while focusing educational interventions to address a learner’s understanding of each category and theme will be important for improving resident education in this area of knowledge and practice.

Process was the theme that contributed most significantly to our understanding of the ontological framework, specific tools, and language needed to function within the handoff procedure. Knowledge of clinical emergency medicine vocabulary and the ability to present this information in a format others understood were the two requirements to participate in a handoff. However, in analyzing provider perceptions, it became apparent that the order in which information is given, and thus conceptualized, cannot be divorced from the expectations and environmental factors influencing it. These factors included cultural phenomena (such as individual preferences, individual skill in story-telling, practice and expertise over time); the ontological framework in which the process of handoff is grounded (the assumed knowledge of medical terminology and the language in which action–tests, labs, prognosis, etc.,–is couched in); and the environmental realities of working in a fast-paced, high-volume setting. These factors form a collective conceptual understanding of the general ED setting that influence the type of information passed along and in what manner. An individual giving handoff draws from this information set in order to pass along information to the oncoming team and thus help form individual conceptual understanding. Therefore, providers expressing frustration with lack of order are actively calling upon, and critiquing, this foundational background that shapes how the information is being relayed to them. The other themes identified from provider perceptions inform how this process of collaboration and consensus building plays out and to what extent it is efficacious.

The factors identified in the Environment theme were consistent with previously reported factors that impact ED handoffs (location, interruptions, ambient noise level, etc).^1^,^7^ While the physical layout of the ED may make some of the environmental factors more or less of an issue for handoffs, we did not identify any factors that were not previously reported or unique only to this academic ED. Still, handoff location was perceived as not only the background for ED handoff culture, but also a direct influence on how providers interacted with each other within that culture. Handoff location plays a central role, both positive and negative, in interprofessional relationships, as the site of both collaboration and relationship formation. Additionally, location, and perceptions of duties in the context of specific locations, influences the efficacy of collaboration and consensus building between providers. This can be seen in the perception of interprofessional interactions between nursing and resident/attending providers; depending on the location, these moments have the potential to disrupt collaboration (i.e., interruptions), or allow for further consensus building and learning. Cultural expectations may potentially evolve at locations that bring together patients and interprofessional providers. Location is not simply a utilitarian factor, a shield from the surrounding chaos, but also a potential barrier, or facilitator, of effective education and assessment.

The themes of Culture and Time, and subthemes of cultural expectations and accepted practices identified by our team all have the potential to impact Process. Individuality, hierarchies and differences in expectations between various roles participating in the handoff were significant subthemes identified, and educational interventions designed to teach and assess handoff performance can avoid related barriers by defining roles and setting standard expectations. Additionally, the relationship between culture and location, explored above, was an important interprofessional subtheme identified. Although the “ideal” setting for handoffs is unknown, assessing interprofessional perceptions of handoff locations that best facilitate collaboration is an important precursor to implementing handoff educational interventions. Finally, many of the environmental barriers to optimal ED sign-out may only be minimized, rather than eliminated. Likewise, the ontological framework and language in which educational interventions take place may be difficult to alter due to the larger culture of the medical system. Educators designing interventions to teach, assess, and ultimately improve ED handoffs might prioritize efforts to change the underlying culture, as the effectiveness of these interventions may rely less on specific procedural changes and more on how they change cultural expectations, perceptions, and norms.

## LIMITATIONS

A potential limitation of this qualitative study is that we performed purposive sampling from a single center. However, we asked participants to rely upon their experiences at all prior settings when answering questions regarding handoff perceptions, and therefore believe the perceptions represent multiple ED settings. Although efforts were made to ensure thematic saturation and data credibility, it is possible there are additional relevant themes that were not uncovered by our study. Finally, although the sampling and FG structure were purposive to facilitate interprofessional discussion, it is possible additional themes would have been uncovered if groups were stratified by discipline.

## CONCLUSION

Interprofessional ED providers in this qualitative study identified four major categories that contribute to the collective conceptual understanding of the ED provider handoff. Understanding this framework and the themes that create it has important implications for emergency medicine resident education. Educators wishing to develop educational interventions to improve resident education in emergency medicine handoff knowledge and practice may wish to explicitly consider how the intervention may impact and interact with these factors, as they may affect learners’ acceptance and incorporation of the intervention.

## Figures and Tables

**Figure:**
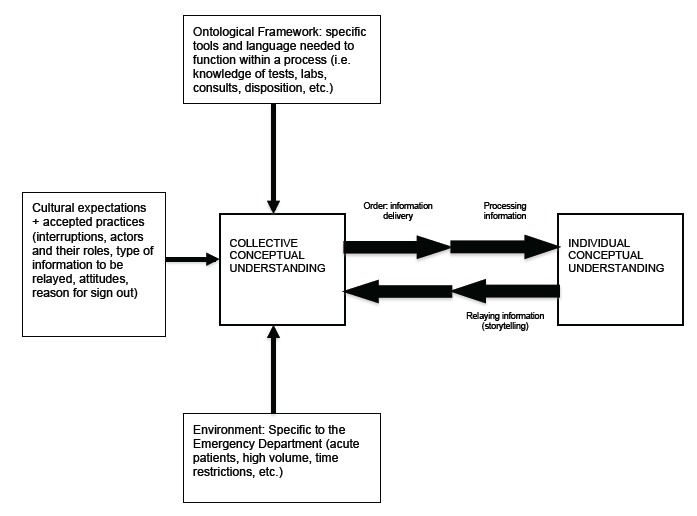
Individual and collective understanding in consensus building processes.

**Table:** Themes, subthemes, representative quotes, and educational considerations with regard to teaching and assessing handoffs in the emergency department.

Themes	Representative quotes	Educational considerations
Culture		
Individuality and educational interventions	“This is why it’s important for it not be standardized. Because when your standardized sign-out is given to [one attending], and that’s not the way he likes it, then you have to follow your [standardized way] and that’s not he how he likes it.”^17^	Importance of developing faculty support for the standardized handoff process
Perceptions of cultural power dynamics and educational interventions	“It’s not standard. Some attendings take over all the tasks, some residents take over all the tasks, it just depends on how they decide to do it… It can be vague. You can leave the sign out process and not be sure who is going to do what.”^19^ “There have been some [attendings] who are more like the, ‘you’re here to take sign out from me, so you come find me, not the other way around’… And I hope that’s an attitude that’s not too prevalent.”^18^	Teach and practice closed loop communication between resident and attending. Set clear departmental guidelines for resident and attending responsibilities of tasks after handoff.
Perceptions of new systems, learning, and educational interventions	“It’s very dependent on who’s giving the sign-out. Because we get more efficient as we mature through this career. So, an intern may be a little too verbose and add detail that’s not important, or they may kind of forget a few of the important things.”^18^ “It, again, is… how good you are at signing out and really honing in on pertinent things.”^18^ “I think in general we’ve all learned a certain way in medicine that we like to hear things based on, obviously this is a more succinct presentation, but it just helps to tell a story, and I think during sign-out a lot of people throw that all out the window and are all over the place.”^17^ “It’s like, it’s like a recipe for making cookies, right? You can have a great recipe and still eff it up.”^18^	Education on how to present a handoff in the ED setting and clinical simulation or case based practice sessions. Importance of having a standardized way to present a handoff in order to teach resident learners. Feedback from senior residents and attendings on how to improve handoff presentation.
Changing systems and improved outcomes	“But I really strongly feel that if we changed the expectations…what is appropriate to be done surrounding sign-out, then we can use our department effectively, and we can do it anywhere in the department if there is clear communication about what’s going on.”^17^ “Less misses. It’s, I mean, the spirit of a sign-out really is mostly a safety issue, not an efficiency issue, though it would be ideal to be efficient at the same time. But I think that a sign-out is for safety.”^18^	Education on time management of ED patient load and preparing for handoffs. Set clear departmental guidelines for what tasks should not be signed out.
Location		
Provider perception of specific sign-out location	“I think there’s a lot of interruptions because of the location.”^17^ “It’s loud. It’s loud for the patients. It’s loud for the doctors. And it lends itself to interruptions.”^19^	Designated sign-out locations perceived as necessary to effective hand-off process.
Provider perception of sign-out location and interprofessional relationships	“I’m not sure if any of you were here when the doctors used to be in [a dedicated room], that was where they sort of lived, and it was not good in that the nurses weren’t free to go in there and ask questions and they were separated.. I think [a dedicated room] is dangerous…. Because there are people who will not come out of there.”^19^ “Well for me, because I’m new, it’s nice to be able to ask that attending face to face.”^18^ “It doesn’t make sense for all of the other nurses come around and the department come to a grinding halt when you’re scheduling patients all over the department.”^17^	Recognition of sign-out location affecting both how providers interact/learn from each, as well as how they learn about and interact with patients. Efficacy of educational intervention tied to space.
Provider perception of sign-out location and provider-patient relationships	“I think patients and families would be way more satisfied if we handed-off in the room. Because how many times do we say ‘Okay Mr. Smith, oh you got tummy pain? We’re going to do all these things here, and we’ll come let you know when they’re done’. And then you go home, and then some stranger comes in, and they’re like, ‘Who are you?’. Like, ‘Oh, I’m Dr. So-and-So, taking over for So-and-So, and I heard so-and-so, and this is that, and the other thing.’”^18^ “Where I trained we did bedside report, and… I think it cut down on a lot of error... And I always try to meet the patient … I think it’s better. Because then you physically lay eyes on the patient. And I know it’s hard … but at the same time, not looking at the patient happens too much.”^19^	Bedside handoffs may provide a different level of safety for learners to practice handoff skills than provider-only locations
Themes	Representative quotes	Educational considerations
Process		
Provider perception of process and order	“Okay, unless this is just how my mind works as I’m coming onto sign-out, the things that I want to hear.. in this order … up front, is this the sickest patient and/or if this is potentially the sickest patient that I need to worry about because it immediately changes my way of thinking.”^17^ “[The action list] is kind the heart of the sign out, ‘cause it’s like ‘I couldn’t get this done, so you get it done for me’ and then we have a disposition.”^18^ “It’s sort of my pet peeve to go out of order in sign outs… I can’t follow that.”^17^	Importance of standardized process
Provider perception of process, order, collective understanding, and consensus building	“[Standardization is] just predictable, and it’s the same every time… I mean, maybe not, you can’t do it every time exactly identical, but if we have the same format, then everyone is getting the information they need.”^18^ “So, when they’re going all over the place, I can’t really chart in order… it just helps to tell a story, and I think during sign-out a lot of people throw that all out the window and are all over the place.”^19^ “I want to know what to concentrate on. And when she tells me, ‘oh, you have a patient in 5 that’s kind of sick and one in 12 that’s kind of sick’, then I know then to keep an eye on those two and make sure that they’re being taken care of.”^18^ “so the other person doesn’t have to find you… so the other person doesn’t have to reinvent… what the problem already is.”^19^ “So making sure that you like spoon feed the critical though process to the next team, and you hope that they rethink it, but they might not, so.”^19^ “It’s [synthesis] is probably really good, because then you know that the person that gave it to you, like, took the key points and was able to kind of succinctly throw it back at you.”^19^ “So, like, so that I can sort of pick up where they left off, in terms of what was the hold up, what was the problem we saw, and be directed by someone who’s been there for twelve hours versus scrambling around trying to find it myself. So having like, you know, where’s the first place that you should go.”^19^ “if there’s a vague plan, that has been passed on, that seems that vagueness spirals into badness.”^19^	Conceptual understanding of a system as two-fold; the individual provider and the collective group or culture. Information order – influence by cultural expectations, ontological frameworks, and the ED environment – and consensus building through storytelling link these two levels, emphasizing closed-loop communication in educational and assessment processes.

*ED,* emergency department
